# Bacterial and fungal colonization and decomposition of submerged plant litter: consequences for biogenic silica dissolution

**DOI:** 10.1093/femsec/fiw011

**Published:** 2016-01-19

**Authors:** Hanna Alfredsson, Wim Clymans, Johanna Stadmark, Daniel Conley, Johannes Rousk

**Affiliations:** 1 Department of Geology, Geocentrum II, Lund University, SE-223 62 Lund, Sweden;; 2 Department of Microbial Ecology, Ecology building, Lund University, SE-223 62 Lund, Sweden

**Keywords:** bacteria, dissolution, fungi, phytoliths, plant litter, silica, remineralization

## Abstract

We studied bacterial and fungal colonization of submerged plant litter, using a known Si-accumulator (*Equisetum arvense*), in experimental microcosms during one month. We specifically addressed the microbial decomposer role concerning biogenic silica (bSiO_2_) dissolution from the degrading litter. To vary the rates and level of microbial colonization, the litter was combined with a range of mineral nitrogen (N) and phosphorous (P) supplements. Overall microbial growth on plant litter increased with higher levels of N and P. There was a tendency for higher relative bacterial than fungal stimulation with higher nutrient levels. Differences in microbial colonization of litter between treatments allowed us to test how Si remineralization from plants was influenced by microbial litter decomposition. Contrary to previous results and expectations, we observed a general reduction in Si release from plant litter colonized by a microbial community, compared with sterile control treatments. This suggested that microbial growth resulted in a reduction of dissolved Si concentrations, and we discuss candidate mechanisms to explain this outcome. Hence, our results imply that the microbial role in plant litter associated Si turnover is different from that commonly assumed based on bSiO_2_ dissolution studies in aquatic ecosystems.

## Abstract (precis)

Rather than enhancing dissolved silicon release during litter decomposition (a common anticipation), microbes can reduce the rate of release of dissolved silicon.

## INTRODUCTION

Microorganisms are the principal regulators of biogeochemical cycles (Rousk and Bengtson 2014). In terrestrial ecosystems, a large fraction of the net primary production enters the soil detrital pool (Cebrian 1999). Here, bacteria and fungi dominate the decomposition of detrital matter (e.g. plant litter) (Gessner *et al.*^2010^; Schimel and Schaeffer 2012). Microbial colonization of plant litter and its decomposition rate is strongly influenced by nutrient availability (e.g. nitrogen (N) and phosphorous (P)) (Scheu 1993; Henriksen and Breland 1999; Aldén, Demoling and Bååth 2001; Sistla, Shinichi and Schimel 2012) along with substrate carbon (C) quality (Högberg, Högberg and Myrold 2007; Strickland *et al.*^2009^; Rousk and Frey 2015). In addition, the same factors also influence the balance between fungal and bacterial colonizers (Gulis and Suberkropp 2003a,b; Mille-Lindblom, Fischer and Tranvik 2006; Rousk and Bååth 2007; Güsewell and Gessner 2009). The separate nutrient acquisition strategies (DeBoer *et al.*^2005^) and flexibility in nutrient homeostasis between fungi and bacteria (Sterner and Elser 2002) are thought to result in different propensities for nutrient release during litter degradation (Güsewell and Gessner 2009; DeVries *et al.*^2011^). As such, the fungal to bacterial balance can characterize the biogeochemical consequences of litter degradation, thus, defining whether the nutrients held in litter will result in nutrient leaching (previously associated with bacterial dominance) or retention in the microbial biomass and subsequent soil sequestration (previously associated with fungal dominance) (DeVries *et al.*^2011^; Schmidt *et al.*^2011^; Clemmensen *et al.*^2013^).

While the interactions between nutrient availability, microbial colonization and release of nutrients during decomposition of plant litter have been studied for e.g. C, N and P, other plant material constituents, such as silicon (Si), have not received explicit study. Si is considered a ‘beneficial’ element for plants providing structural support (Schoelynck *et al.*^2010^), protection against herbivory (Massey and Hartley 2006; Massey, Ennos and Hartley 2006) and alleviation against biotic and abiotic stressors (Ma and Yamaji 2006, 2008). Dissolved Si (DSi: H_4_SiO_4_) taken up by plants precipitates as amorphous Si (SiO_2_ × *n*H_2_O) into the biomass which is commonly termed biogenic Si (bSiO_2_) (Epstein 1994). The extent of Si uptake varies among plant species but grasses (Poaceae), sedges (Cyperaceae) and horse-tails (Equisetaceae) represent known Si-accumulators (Hodson *et al.*^2005^). Through litterfall, bSiO_2_ accumulates in soil and this pool is commonly larger than what is stored in aboveground biomass (Blecker *et al.*^2006^).

Based on their role as primary decomposers of soil organic matter it has been anticipated that microbial decomposition also will influence Si release from plant litter (Clarke 2003; Sommer *et al.*^2006^; Schoelynck *et al.*^2010^; Struyf and Conley 2012; Schaller and Struyf 2013). Microbial decomposition is known to accelerate bSiO_2_ dissolution (diatom frustules) in pelagic (Bidle and Azam 1999, 2001; Bidle, Manganelli and Azam 2002; Roubeix, Becquevort and Lancelot 2008) and benthic (Holstein and Hensen 2010) ecosystems. The mechanism by which aquatic bacteria accelerate diatom bSiO_2_ dissolution is through ectoenzymatic decomposition of an outer organic coating (Patrick and Holding 1985; Bidle and Azam 2001). However, to date the question still remains open whether microbial decomposers in terrestrial ecosystems can enhance Si release from phytoliths embedded in an organic matrix. Struyf and Conley (2012) identified that one of the key aspects for prioritized study should be to characterize the ‘terrestrial ecosystem filter’, where cycling of Si within the plant–soil system directly alter Si fluxes through the land–ocean continuum (Derry *et al.*^2005^; Carey and Fulweiler 2013).

The few available studies designed to investigate dissolution of bSiO_2_ during microbial decomposition of plant litter show contradictory results, and they were not designed to explicitly investigate the role of microorganisms. Struyf *et al.* (2007) incubated reed (*Phragmites australis*) litter in river water factorially with a broad spectrum bacterial antibiotic. While there was a tendency for slight reductions of Si release in the presence of antibiotics, the influence of bacteria was concluded to be negligible. Similarly, Fraysse *et al.* (2010) incubated different types of plant litter in aqueous solution where some of the plant material was sterilized by autoclavation. The microbial decomposer community resulted in 2.5–10 times higher Si release compared with sterile experimental systems. However, both Struyf *et al.* (2007) and Fraysse, Pokrovsky and Meunier (2010) assessed the influence of an actively degrading microbial community indirectly, without verifying the presumed difference in colonization between control and inhibited or sterilized samples. Additionally, Fraysse, Pokrovsky and Meunier (2010) did not distinguish between fungal and bacterial decomposers while Struyf *et al.* (2007) selectively inhibited bacteria with unknown fungal responses. As such, the relative contribution of bacteria and fungi to Si release from plant litter remains unassessed. Investigating the effect of microbial colonization and decomposition on Si release would elucidate (i) the role of biological factors in regulating Si release into soil pore water and (ii) how Si fluxes are influenced by the rate and type of microbial decomposition of submerged plant litter.

The objective of this study was to investigate how the rate (degree of colonization) and type of (fungal or bacterial) microbial use of plant litter influenced bSiO_2_ dissolution. We selected *Equisetum arvense*, a known Si-accumulating plant (Hodson *et al.*^2005^) with high susceptibility to microbial degradation (Marsh *et al.*^2000^), for this investigation. We postulated a set of hypotheses as follows. (H1) The rate of Si release from submerged litter would increase with the level of microbial colonization. Mineral nutrient supplements (N and P) added with submerged litter was used to increase the level of microbial colonization. (H2) Higher levels of mineral nutrient supplements to submerged litter would shift the colonizing microbial community toward a bacterial dominance. (H3) A shift toward a higher fungal dominance of the colonization and decomposition of submerged plant litter would yield a lower rate of Si release due to generally more nutrient retentive strategies through flexible homeostasis. We addressed these hypotheses by incubating Si-rich plant material in aquatic microcosm systems in the presence of or absence of microorganisms in laboratory conditions during ∼1 month time. Nutrient supplements (N and P) were added at four different levels to achieve differences in the degree of microbial decomposition of plant litter, allowing us to study its effect on Si release. Bacterial growth and Si release were monitored at high temporal resolution while fungal abundance was estimated for the end-point.

## MATERIALS AND METHODS

Two separate microcosm experiments were conducted to assess microbial colonization of plant litter and the effect of litter decomposition on Si release. First, in ‘Experiment I’, we compared the potential influence of two different sterilization techniques, along with the influence of a live microbial community on Si release from plant litter. In this system, a bacterial inoculate was used, and only the bacterial growth on the submerged litter was studied. The degree of microbial colonization increased over time, and bacterial growth and Si release were monitored. Second, in Experiment II, we investigated the influence of nutrient availability (N and P) on bacterial and fungal colonization of submerged plant litter and how differences in microbial colonization and decomposition degree influenced release of Si. In this experiment, a microbial inoculum including both fungi and bacteria was used, and the degree of bacterial growth and Si release were monitored over time and the fungal colonization was assessed at the end of the incubation.

### Preparation of plant litter

For both Experiments I and II, horse-tails (*E. arvense*) were collected from the shore of a small pond (Lund, Sweden) at the end of the growing season (August 2013 and September 2014, respectively). All plant material was washed with Milli-Q water (MQ) to remove dust and other particles.

For Experiment I, cleaned and fresh *E. arvense* were homogenized into ∼1 cm pieces. The fresh plant material was packed into foil and sterilized. Two methods for sterilization were tested where autoclaving for 20 min was compared to heating at 80°C for 12 h. This was done to evaluate the efficiency of the applied sterilization method and its potential impact on Si release from colonized plant material. Each method of sterilization was performed three times with the plant material stored sterile in foil at room temperature for ∼72 h to ensure sterilization of any germinated spores. After sterilization, plant material was kept refrigerated until use in the experiment.

For Experiment II, cleaned *E. arvense* were dried at 40°C and homogenized into ∼1 cm pieces. The moisture of plant material was adjusted with MQ water, packed into foil and sterilized by autoclaving (40 min). Autoclaving was performed twice with the plant material stored sterile in foil at room temperature for ∼72 h in between (see above). The plant material was kept refrigerated until use in the experiment.

### Preparation of microbial inoculum

For both Experiments I and II, surface soil (0–2 cm) collected from the same site as the *E. arvense* were used as a microbial inoculum. For Experiment I, fresh soil (4 g) was mixed with 20 mL of autoclaved MQ water (0.2 g soil mL^−1^) and vortexed for 3 min. The soil slurry was gravity filtered twice through a 1 μm (47 mm) polycarbonate filter (Nucleopore), found to exclude fungi (Fægri, Torsvik and Goksöyr 1977; Møller, Miller and Kjøller 1999; Mille-Lindblom and Tranvik 2003). Half of the filtered inoculum was autoclaved twice (henceforth referred to as ‘sterile inoculum’) following the same procedures described for the plant material. The other half of the filtered inoculum was kept refrigerated until use in the experiment (henceforth referred to as ‘live bacterial inoculum’).

For Experiment II, fresh soil was sieved through a 2 mm mesh and gently homogenized in a plastic bag. Two hundred milligrams of fresh soil were put into 1.5 mL plastic Eppendorf tubes (one per incubation) of which half was kept cool (henceforth referred to as ‘live microbial inoculum’) until use in the experiment. The other half was autoclaved (henceforth referred to as ‘sterile inoculum’) twice in the same way as described for the plant material.

### Experimental design

All laboratory microcosm experiments were set up in plastic 1 L sterile Erlenmeyer flasks equipped with lids with a hydrophobic filter (0.22 μm) enabling sterile air exchange. Incubations were kept dark and at room temperature (22°C–23°C) for 23 (Experiment I) and 27 (Experiment II) days, respectively.

In Experiment I, sterile plant material (4 g dry weight) was suspended in 950 mL of sterile 0.01M CaCl_2_ (pH 5.8 ± 0.11) followed by inoculation with either 1 mL of a sterile inoculum or a live bacterial inoculum. In total, 12 microcosm experimental units were set up where each treatment was replicated three times (Table 1). During the experiment samples were collected from the aqueous phase for determinations of bacterial growth (1.5 mL) and DSi concentrations (5 mL) during day 0, 1, 3, 4, 8, 11, 16 and 23. The pH (15 mL) was measured during day 0, 8, 16 and 23.

**Table 1.** Overview of experimental design and initial conditions.

Incubation	Sterilization method—plant	Microbial	C:N:P	Corresponding N and P	Starting DSi concentration
no.	material	inoculum	(molar)	concentration (mM)	(μ mol Si g^−1^ dry litter)
*Experiment I*					
1–3	Heated to 80°C, 12h	Sterile	–	–	36 ± 1.8
4–6	Autoclavation, 20 min	Sterile	–	–	46 ± 1.8
7–9	Heated to 80°C, 12h	Live	–	–	30 ± 1
10–12	Autoclavation, 20 min	Live	–	–	48 ± 0.5
*Experiment II*					
1–3	Autoclavation, 40 min	Sterile	200:1:1	1.45	5.8 ± 1.9
4–6	Autoclavation, 40 min	Sterile	100:1:1	2.9	6.4 ± 1.1
7–9	Autoclavation, 40 min	Sterile	50:1:1	5.8	5 ± 0.14
10–12	Autoclavation, 40 min	Sterile	25:1:1	11.6	9.5 ± 0.86
13–15	Autoclavation, 40 min	Live	200:1:1	1.45	5.3 ± 1.6
16–18	Autoclavation, 40 min	Live	100:1:1	2.9	6.4 ± 0.71
19–21	Autoclavation, 40 min	Live	50:1:1	5.8	7.3 ± 3.4
22–24	Autoclavation, 40 min	Live	25:1:1	11.6	8.4 ± 1.9

Data are mean (*n* = 3) ± standard error (SE).

The initial bSiO_2_ content of *E. arvense* litter was 41.7 ± 4.7 and 33.7 ± 1.3 mg SiO_2_ g^−1^dry weight in Experiment I and II, respectively. The bSiO_2_ content of the added soil (i.e. microbial inoculum) was 5.1 ± 0.5 mg SiO_2_ g^−1^ dry weight. Hence, in total 236 ± 9.0 and 1.03 ± 0.10 mg of bSiO_2_ were added to each microcosm from the plant material and soil inoculum, respectively. For Experiment I, the first sampling for measurements of DSi concentrations (i.e. starting DSi concentration) were taken ∼2.5 h after the experiments start, while for Experiment II the first sampling was conducted immediately after starting the microcosms. The bSiO_2_ added from the soil (i.e. microbial inoculum) represents 0.4% of the total bSiO_2_ (*E. arvense* litter + soil) added per microcosm and is considered insignificant.

In Experiment II, sterile plant material (7 g dry weight) was suspended in 1 L of autoclaved (20 min) MQ water amended with N (NH_4_NO_3_) and P (K_2_HPO_4_/KH_2_PO_4_, 50:50 molar basis) supplements at four different levels. These correspond to final C:N:P molar ratios of 200:1:1, 100:1:1, 50:1:1 and 25:1:1 and are representative of C:N ratios found in plant tissue and senesced litter (McGroddy, Daufresne and Hedin 2004; Yang and Luo 2011). In total, 24 batch microcosm experimental units were set up where half was inoculated with 200 mg sterile soil (sterile inoculum) and the other half inoculated with 200 mg non-sterilized soil (live microbial inoculum). Both sterile and live incubations were amended with N and P at the four levels described above. All treatments were replicated three times (Table 1). During the experiment samples were collected from the aqueous phase for determination of bacterial growth (1.5 mL) and DSi concentrations (8 mL) during days 0, 1, 2, 5, 7, 9, 13, 16, 21 and 27. The pH (15 mL) was measured during days 2, 21 and 27. Electrical conductivity (EC) and total organic carbon were measured during day 27. At day 27, the remaining plant material was collected by filtering into ceramic cups (≥1 mm particle retention) and used for estimation of fungal abundance by extracting ergosterol.

### Bacterial growth and fungal abundance

Bacterial growth was estimated by Leucine incorporation (Kirchman, K'Nees and Hodson 1985) with modifications (Rousk, Brookes and Bååth 2009), which estimates the rate of protein synthesis as a measure of bacterial growth using a 1 h incubation at 22°C without light. Non-incorporated [^3^H] Leu was removed from samples by serial washing (Bååth, Pettersson and Söderberg 2001), and the incorporated radioactivity was determined to estimate the leucine incorporation rate.

To also retrieve bacteria growing as biofilms on the plant material, three similar sized plant straws were transferred from respective incubation into the 1.5 mL liquid sample aliquot (three technical replicates per incubation) at day 27. The samples were sonicated for 5 min in a water bath followed by immediate [^3^H]Leu incorporation assays (see above).

Fungal abundance was estimated by extracting the membrane lipid ergosterol from freeze dried and homogenized plant litter collected at day 27 of Experiment II. To estimate initial concentrations in the plant material before the experiment started, ergosterol was extracted from the original plant litter (six replicates). Ergosterol was analyzed as previously described (Rousk and Bååth 2007; Rousk, Brookes and Bååth 2009).

### Amorphous and dissolved Si

The bSiO_2_ content of *E. arvense* litter at the start of experiment and the soil used for microbial inoculum (Experiment II) was determined by wet alkaline digestion in 1% Na_2_CO_3_ as previously described (DeMaster 1981; Saccone *et al.*^2007^) using a sample weight of 30 mg. Extracts were colorimetrically analyzed for DSi (SmartChem 200) with the molybdate-blue methodology.

The increase in DSi concentrations over time was used to study bSiO_2_ dissolution from *E. arvense* litter. Subsamples (8 mL) were collected from the aqueous phase and filtered through 0.45 μm Sterivex filters (Millipore) into plastic tubes. Samples were stored refrigerated until analysis. Samples were analyzed for DSi with the molybdate-blue methodology in Experiment I. In Experiment II DSi samples were analyzed by Inductively coupled plasma—atomic emission spectroscopy (ICP-AES; Thermo ICAP 6500 duo, Thermo Fisher) since the addition of P to microcosms interfered with the determination of Si using the molybdate-blue methodology.

### pH, electrical conductivity, TC, IC and TOC

The pH was monitored but not controlled during both experiments. For pH, 15 mL subsamples (unfiltered) were collected from the aqueous phase, and the pH was measured using a pH-meter. EC was measured with a conductivity meter. TC, IC and total organic C (TOC) were measured on unfiltered samples using a total organic carbon analyzer (Shimadzu TOC-V CPN).

### Statistical analysis

Statistical analysis of data was performed in Graph Pad Prism 6. All data were log transformed prior to statistical testing to stabilize variances. Differences between means were analyzed using *t*-test, ANOVA and Two-way ANOVA. If significant differences were detected with ANOVA, differences between treatments were examined by Tukey's post hoc tests (α = 0.05). The dependence of DSi on microbial degradation was tested using a type-II major axis regression.

## RESULTS

### Bacterial growth

**Figure 1.**
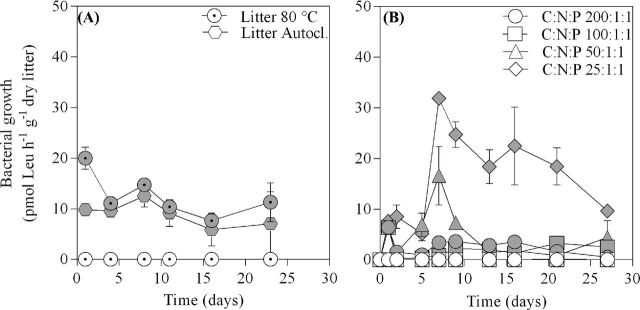
Symbols represent mean values ± 1 standard error (*n* = 3) of bacterial growth rate over time estimated by [^3^H]Leu incorporation for the different treatments in (**a**) Experiment I and (**b**) Experiment II. Treatments in Experiment I include litter sterilized at either 80°C for 12 h or by autoclavation while in Experiment II treatments include N and P amendments at four different levels (C:N:P 200:1:1, 100:1:1, 50:1:1 and 25:1:1) that have been factorially treated with a live (filled symbols) or sterile (open symbols) inoculum.

**Figure 2.**
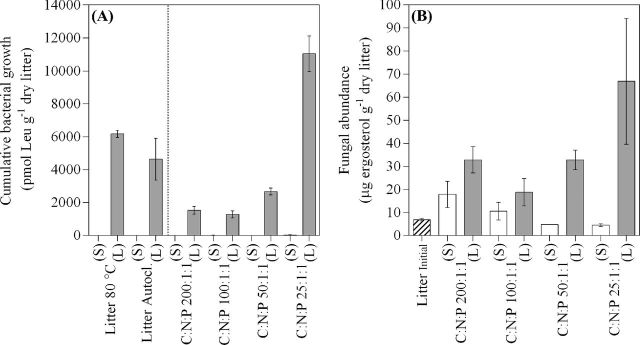
Mean values ± 1 standard (*n* = 3) of (**a**) cumulative bacterial growth for Experiment I and II and (**b**) fungal abundance for the different treatments in Experiment II at the end of the experiment (after 23 or 27 days for Experiment I and I, respectively). Note that cumulative bacterial growth in sterile microcosms is too low to show on the graph. Treatments in Experiment I include litter sterilized at either 80°C for 12 h or by autoclavation while in Experiment II treatments include N and P amendments at four different levels (C:N:P 200:1:1, 100:1:1, 50:1:1 and 25:1:1) that have been factorially treated with a live (L, filled bars) or sterile (S, open bars) inoculum.

Initial bacterial growth rates in Experiment I were highest in live microcosms supplied with plant litter heated to 80°C (∼20 pmol Leu h^−1^ g^−1^ dry litter) when compared to autoclaved plant litter (∼10 pmol Leu h^−1^ g^−1^ dry litter). After day 1, however, bacterial growth rates converged (at ∼10 pmol Leu h^−1^ g^−1^ dry litter) in the two treatments and remained similar until the end of experiment (Fig. 1a). This resulted in no significant difference in cumulative bacterial growth between the two treatments at the end of the experimental period (*t*-test, *P* = 0.25; Fig. 2a).

In live microcosms of Experiment II, bacterial growth increased immediately reaching a similar level during day 1 in all treatments (∼7 pmol Leu h^−1^ g^−1^ dry litter). The bacterial growth in lower nutrient treatments (C:N:P 200:1:1 and 100:1:1) then declined by day 2–3 and remained relatively low (∼2–3 pmol Leu h^−1^ g^−1^ dry litter). In microcosms amended with higher N and P amounts (C:N:P 50:1:1 and 25:1:1) bacterial growth rates increased to a maximum rate at about day 7 (∼17 and ∼32 pmol Leu h^−1^ g^−1^ dry litter, respectively). The bacterial growth rates at day 7 increased with higher nutrient supplements (C:N:P 25:1:1 = 50:1:1 > 200:1:1 > 100:1:1; ANOVA, *P* < 0.0001; Fig. 1b). Bacterial growth rates in treatment C:N:P 50:1:1 then converged to levels similar to C:N:P 200:1:1 and 100:1:1, while bacterial growth in the C:N:P 25:1:1 treatment remained one order of magnitude higher for the duration of the experiment. This resulted in a cumulative bacterial growth that differed between treatments (C:N:P 25:1:1 > 50:1:1, 100:1:1, 200:1:1, C:N:P 50:1:1 > 100:1:1; ANOVA, *P* = < 0.0001; Fig. 2a).

In Experiment I, sterile microcosms remained free from bacterial growth during the whole experiment (Figs 1a and 2a). Most sterile microcosms remained free of microbial growth in Experiment II but bacterial growth was gradually noticed in up to half of the sterile microcosms from day 16. However, the bacterial growth was still well below that in live microcosms (Fig. 1b) and at the experiment's final day cumulative bacterial growth remained ≤1% of that in live incubations for all treatments (Fig. 2a).

### Fungal abundance and fungal-to-bacterial ratios

**Figure 3.**
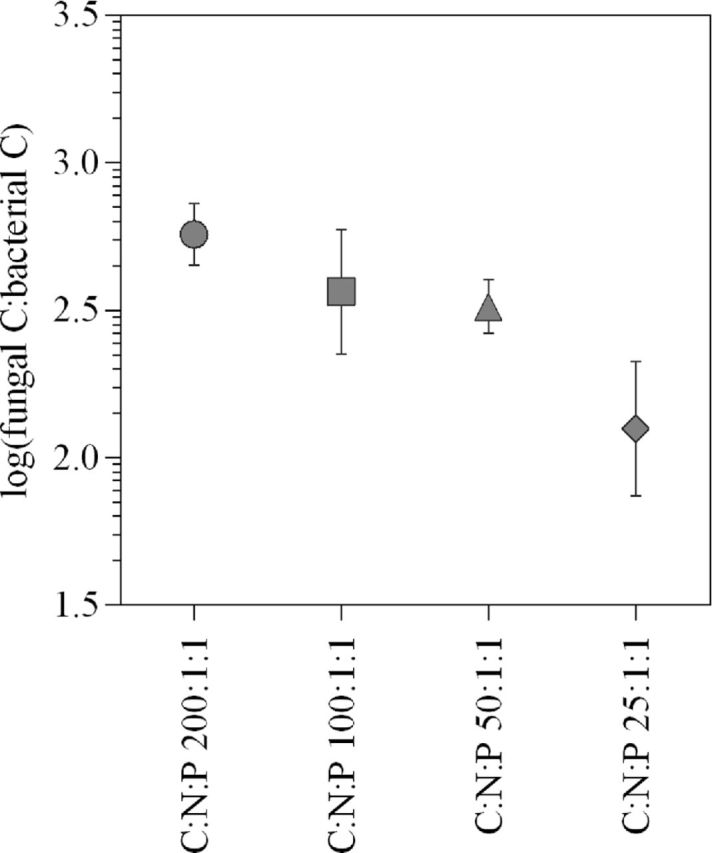
Relationship between fungal abundance and cumulative bacterial growth at the end of the experiment for the different nutrient treatments under live conditions in Experiment II. The symbols represent mean values ±1 standard error (*n* = 3). Experiment II treatments include N and P amendments at four different levels (C:N:P 200:1:1, 100:1:1, 50:1:1 and 25:1:1) combined with a live inoculum.

In live microcosms of Experiment II, fungal abundance ranged between 19–67 μg ergosterol g^−1^ litter at the experiments final day. Compared to an initial content of 6.8 ± 0.62 μg ergosterol g^−1^ litter (mean ± SE), the ergosterol content had increased significantly (ANOVA, *P* < 0.0003; Litter C:N:P 200:1:1, 50:1:1 and 25:1:1 > Litter_initial_) showing that fungal growth had occurred in the microcosms. Although not significant (*P* = 0.25), the fungal response to increasing nutrient supply (Fig. 2b) was similar to that observed for bacteria (Fig. 2a, Experiment II). This resulted in a trend with decreasing abundance of fungi in relation to cumulative bacterial growth with increasing levels of N and P supply (Fig. 3). However, differences between treatments were not statistically significant (One-way ANOVA, *P* = 0.12).

Sterile microcosms remained largely free from fungal growth (Fig. 2b). Compared to the initial litter content, a significant change (ANOVA, *P* = 0.013) in litter ergosterol content had occurred over the duration of the experiment. This was caused by fungal growth in one of three C:N:P 200:1:1 replicates (visual observation) which was also supported by an elevated ergosterol content (∼30 μg ergosterol g^−1^ litter) in this replicate. However, fungal abundance did not differ between the other sterile treatments compared to the initial content. This indicates that detected amounts originated from ergosterol not destroyed by sterilization rather than from fungal growth during the experiment. Moreover, the well-known propensity for ergosterol to be resistant to degradation (Mille-Lindbom, Von Wachenfeldt and Tranvik 2004; Zhao, Lin and Brookes 2005) implies that final concentrations (in live microcosms) reflected cumulative production during the course of our experiments.

### Si release from plant litter

**Figure 4.**
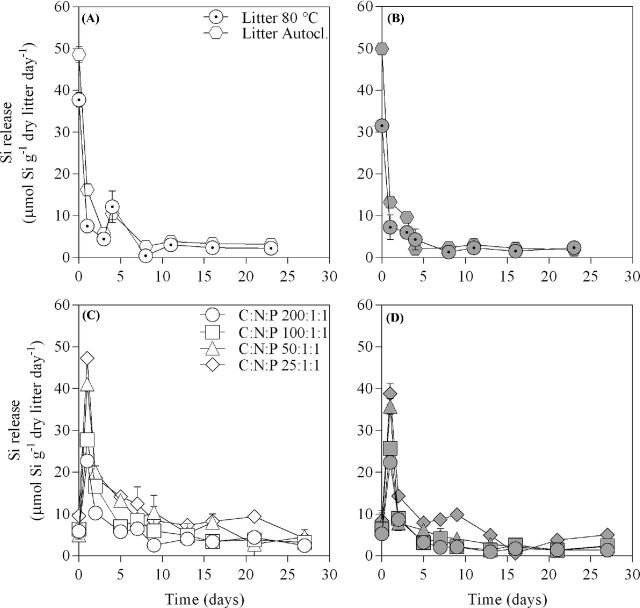
Mean values ±1 standard error (*n* = 3) of Si release rate over time for the different treatments in (**a**) Experiment I under sterile conditions, (**b**) Experiment I under live conditions, (**c**) Experiment II under sterile conditions and (**d**) Experiment II under live conditions. Treatments in Experiment I include litter sterilized at either 80°C for 12 h or by autoclavation while in Experiment II treatments include N and P amendments at four different levels (C:N:P 200:1:1, 100:1:1, 50:1:1 and 25:1:1) that have been factorially treated with a live (filled symbols) or sterile (open symbols) inoculum.

**Figure 5.**
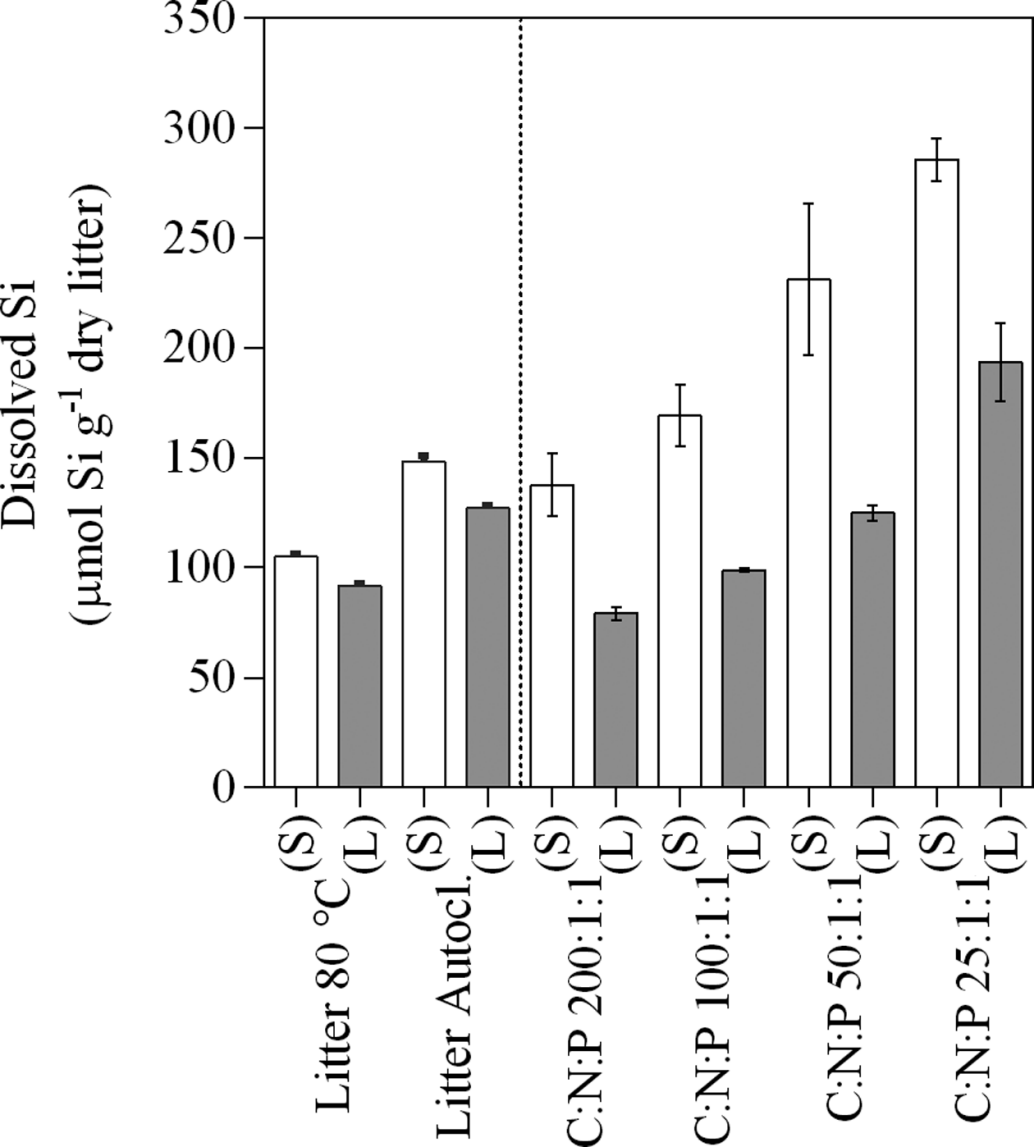
Mean values ±1 standard error (*n* = 3) of DSi concentrations for Experiment I and II for the different treatments. The data are cumulative over 23 or 27 days for Experiment I and II, respectively. Treatments in Experiment I include litter sterilized at either 80°C for 12 h or by autoclavation while in Experiment II treatments include N and P amendments at four different levels (C:N:P 200:1:1, 100:1:1, 50:1:1 and 25:1:1) that have been factorially treated with a live (L, filled bars) or sterile (S, open bars) inoculum.

The dissolution of bSiO_2_ from *E. arvense* litter is described by an initially fast release followed by a progressively slower release of Si in all treatments. Over the course of Experiment I, Si release rates under sterile conditions tended to be overall slightly higher than or similar to those under live conditions independent of method of litter sterilization (Fig. 4a and d). However, accumulation of these statistically indistinguishable differences over time resulted in final DSi concentrations that were significantly higher under sterile compared to live conditions for both types of heat-treated litter (Two-way ANOVA, *P* < 0.0001; Fig. 5.) Moreover, Si release rates were initially higher from autoclaved litter than from litter only heated at 80°C under both sterile and live conditions. The effect of litter treatment gradually became less pronounced toward the end of the experiment (Fig. 4a and b). Though, at the end of Experiment I final DSi concentrations were markedly higher in treatments supplied with autoclaved litter compared to treatments supplied with litter heated at 80°C under both sterile and live conditions (Two-way ANOVA, *P* < 0.0001; Fig. 5).

In Experiment II, initial (day 1) Si release rates varied greatly between treatments (Fig. 4c and d). We found that the inoculum (sterile or live) had an effect on initial (day 1) Si release rates (Two-way ANOVA, *P* = 0.0011). However, the effect was only distinguishable at the highest nutrient supply level (C:N:P 25:1:1) where the initial Si release rates were higher under sterile (Fig. 4c) conditions compared to live (Fig. 4d). Final (day 27) Si release rates in Experiment II did not differ between sterile and live conditions (Two-way ANOVA, *P* = 0.19; Fig. 4c and d). However, over the course of Experiment II, the overall dynamics resulted in final DSi concentrations (Fig. 5) that were significantly higher under sterile conditions compared to live conditions (Two-way ANOVA, *P* < 0.0001) at all nutrient levels. In fact, the presence of a microbial community reduced the release of Si by 30–40% when compared to its sterile control treatment at equal C:N:P level (Fig. 5). Moreover, the N and P treatments had a strong influence on bSiO_2_ dissolution, increasing with higher N and P supply (two-way ANOVA, *P* < 0.0001) under both sterile and live conditions, and its influence was more pronounced than the effect of inoculum (Fig. 4c and d). The effect of N and P additions on Si release rates gradually grew less pronounced over time, but remained distinguishable throughout the experiment (two-way ANOVA, *P* = 0.042; Fig. 4c and d). This resulted in final DSi concentrations (Fig. 5) that were higher with increasing level of N and P supply (Two-way ANOVA, *P* < 0.0001) independent of inoculum.

### Microbial use of litter and Si release

**Figure 6.**
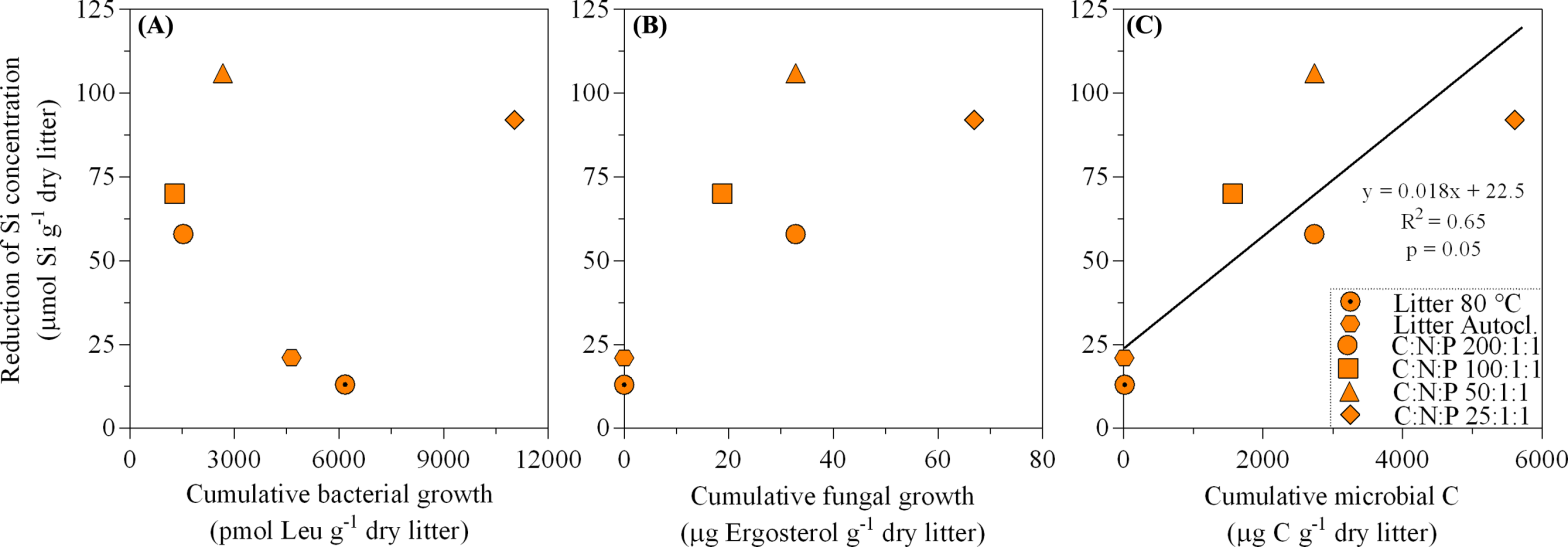
Relationship between the reduction in Si concentration and (**a**) cumulative bacterial growth, (**b**) cumulative fungal growth and (**c**) cumulative microbial carbon (C) for the different treatments in Experiment I and II. The data are cumulative over 23 or 27 days for Experiment I and II, respectively. The reduction in Si concentration represents the difference in DSi in sterile versus live treatments at equal C:N:P level, thus subtracting the DSi at live conditions from the DSi at sterile conditions. Treatments in Experiment I include litter sterilized at either 80°C for 12 h or by autoclavation while in Experiment II treatments include N and P amendments at four different levels (C:N:P 200:1:1, 100:1:1, 50:1:1 and 25:1:1).

To estimate total microbial growth, we used conversion factors to estimate fungal C from ergosterol (Fig. 6b) and bacterial C from leucine incorporation data (Fig. 6a). Estimates of fungal biomass associated with litter (mg fungal C g^−1^ litter) were obtained using a conversion factor of 5.5 mg ergosterol g^−1^ mycelial dry mass (Gessner and Chauvet 1993; Joergensen 2000), or 12 mg ergosterol g^−1^ fungal C. Estimates of bacterial C production were based on a widely used conversion factor from aquatic studies (3.1 ng bacterial C pmol^−1^ Leu (Cole, Findlay and Pace 1988; Rousk and Bååth 2011; Bottomley, Taylor and Myrold 2012). To assess the microbial role in the release of Si from litter, we focused on the difference between live and sterile treatments. Since the sterile treatments always resulted in a higher release of Si (Fig. 5), we subtracted the Si release in live from that in sterile treatments, resulting in ‘reduction of Si concentration’ by microbial activity (Fig. 6a and b). Combining estimates of both bacterial and fungal production suggested that a higher microbial growth resulted in a reduced concentration of DSi in our experiments (*P* = 0.05; Fig. 6c).

### pH, electrical conductivity, TC, IC and TOC

In Experiment I, there was a slight change in pH from 5.8 to 5.1 in sterile conditions while the pH was stable under live conditions (Table 2). In Experiment II, pH increased from ∼5.0 to 6.5 with higher levels of N and P supply under both sterile and live conditions. The EC increased over the experimental period in all treatments, ranging between 1.5 and 2.5 mS cm^−1^ at the final day (Table 2). The EC did not differ between sterile and live microcosms but there was a significant dependence on the level of N and P supply (Two-way ANOVA, *P* < 0.0001) for both sterile and live conditions.

**Table 2.** pH and EC over time in unfiltered aqueous solution for the different experimental treatments.

Incubation	Microbial	C:N:P	pH	Day	Day	Day	Day	Day	Day	EC (mS cm^−1^)	Day
no.	inoculum	(molar)	Day 0	2	8	16	21	23	27	Day 0	27
*Experiment I*											
1–3	Sterile	–	5.8 ± 0.1	–	5.1 ± 0.03	5.1 ± 0.04	–	5.2 ± 0.03	–	–	–
4–6	Sterile	–	5.8 ± 0.1	–	5.2 ± 0.03	5.4 ± 0.07	–	5.3 ± 0.04	–	–	–
7–9	Live	–	5.8 ± 0.1	–	5.4 ± 0.1	5.9 ± 0.04	–	5.9 ± 0.04	–	–	–
10–12	Live	–	5.8 ± 0.1	–	5.4 ± 0.02	5.8 ± 0.2	–	5.6 ± 0.2	–	–	–
*Experiment II*											
1–3	Sterile	200:1:1	–	5.8 ± 0.01	–	–	5.6 ± 0.2	–	5.8 ± 0.2	0.30	1.5 ± 0.02
4–6	Sterile	100:1:1	–	5.9 ± 0.06	–	–	5.7 ± 0.2	–	5.8 ± 0.2	0.61	1.7 ± 0.02
7–9	Sterile	50:1:1	–	6.0 ± 0.01	–	–	6.1 ± 0.2	–	6.1 ± 0.2	1.2	2.0 ± 0.04
10–12	Sterile	25:1:1	–	6.2 ± 0.02	–	–	6.5 ± 0.02	–	6.3 ± 0.1	2.2	2.7 ± 0.1
13–15	Live	200:1:1	–	5.5 ± 0.04	–	–	5.0 ± 0.06	–	5.1 ± 0.1	0.30	1.5 ± 0.03
16–18	Live	100:1:1	–	5.4 ± 0.03	–	–	5.5 ± 0.09	–	5.5 ± 0.1	0.61	1.7 ± 0.04
19–21	Live	50:1:1	–	5.9 ± 0.01	–	–	5.4 ± 0.06	–	5.5 ± 0.08	1.2	2.3 ± 0.03
22–24	Live	25:1:1	–	6.2 ± 0.03	–	–	6.5 ± 0.09	–	6.7 ± 0.2	2.2	2.6 ± 0.05

Data are mean (*n* = 3) ± standard error (SE). The day 0 pH and EC values are measurements on pure 0.01M CaCl_2_ and growth medium (i.e. MQ water amended with N and P), respectively.

Total carbon (TC) concentrations increased over time in all treatments (Table 3). IC content increased over time in live microcosms (Two-way ANOVA, *P* < 0.0001) likely due to microbial degradation of litter generating CO_2_. TOC concentrations ranged between 430–520 μg mL^−1^ and 350–480 μg mL^−1^ in sterile and live incubations, respectively. The differences in final TOC concentrations between treatments varied significantly due to both inoculum (Two-way ANOVA, *P* = 0.007) and N and P supply level (Two-way ANOVA, *P* = 0.017).

**Table 3.** TC, IC and TOC in unfiltered aqueous solution for the different experimental treatments at the initial (day 0) and final day of experiment (day 27).

Incubation	Microbial	C:N:P	TC—initial	TC—final	IC—initial	IC—final	TOC—initial	TOC—final
no.	inoculum	(molar)	(μg mL^−1^)	(μg mL^−1^)	(μg mL^−1^)	(μg mL^−1^)	(μg mL^−1^)	(μg mL^−1^)
*Experiment II*								
1–3	Sterile	200:1:1	1.1 ± 0.2	518 ± 14	1 ± 0.04	3 ± 0.8	0.1 ± 0.2	515 ± 14
4–6	Sterile	100:1:1	1.2 ± 0.3	498 ± 3	1 ± 0.05	2 ± 0.9	0.2 ± 0.3	496 ± 3
7–9	Sterile	50:1:1	1.5 ± 0.5	521 ± 18	1 ± 0.02	2 ± 0.8	0.6 ± 0.5	519 ± 18
10–12	Sterile	25:1:1	1.4 ± 0.3	437 ± 25	1 ± 0.1	3 ± 1	0.3 ± 0.3	434 ± 25
13–15	Live	200:1:1	1.1 ± 0.2	455 ± 18	1 ± 0.04	11 ± 2	0.1 ± 0.2	444 ± 16
16–18	Live	100:1:1	1.2 ± 0.3	410 ± 4	1 ± 0.05	9 ± 1	0.2 ± 0.3	401 ± 4
19–21	Live	50:1:1	1.5 ± 0.5	493 ± 21	1 ± 0.02	11 ± 2	0.6 ± 0.5	482 ± 22
22–24	Live	25:1:1	1.4 ± 0.3	385 ± 49	1 ± 0.1	40 ± 14	0.3 ± 0.3	345 ± 63

Data are mean (*n* = 3) ± standard error (SE).

The initial TC, IC and TOC values are measured on pure growth medium (i.e. MQ water amended with N and P).

## DISCUSSION

### Microbial colonization and decomposition of litter

Bacterial growth in microcosms containing submerged litter followed a pattern where growth reached maxima followed by a decline (Figs 1a and b). Such a pattern is generally observed under both laboratory (Gulis and Suberkropp 2003a,b) and natural conditions (Gulis and Suberkropp 2003c). As per our design, bacterial growth responded positively to increased levels of nutrient (N, P) additions (Figs 1b and 2a) as previously observed by others for both plant litter (Gulis and Suberkropp 2003a,c) and soil (Aldén, Demoling and Bååth 2001) incubation experiments. Highest bacterial growth were reached in microcosms supplied with most N and P (C:N:P 25:1:1) and was sustained during the experimental period (Fig. 1b). The sustained high bacterial growth rates suggest that nutrients were remineralized from the high biomass built in these microcosms (C:N:P 25:1:1). Moreover, the IC concentrations in live microcosms (Table 3) clearly matched with bacterial growth rates (Fig. 1b) during the experiments final day. These lines of evidence together suggest that bacterial growth on the litter was limited by nutrient availability. The addition of nutrients (N and P) relieved this limitation (Fig. 2a), causing higher degrees of plant litter decomposition. As such, this experimental approach successfully enabled Si release from plant litter to be studied at different levels of litter decomposition (H1). The response to increasing N and P availability by the fungal community was similar to that for bacteria (Fig. 2b). This suggests that fungal growth, although showing a higher variability, was also limited by N and P availability in our microcosms. Moreover, the supply of N and P to microcosms tended to affect fungal and bacterial decomposers asymmetrically, with a dominance of fungi that gradually decreased with increasing nutrient amendments. This tendency is consistent with our hypothesis (H2), but not sufficiently strong to confirm it.

Estimates of fungal biomass associated with litter (mg fungal C g^−1^ litter) were obtained using a conversion factor of 5.5 mg ergosterol g^−1^ mycelial dry mass (Gessner and Chauvet 1993; Joergensen 2000), or 12 mg ergosterol g^−1^ fungal C. Fungal biomass in the current experiment ranged between 1563 and 5577 μg fungal C g^−1^ litter after 27 days of incubation. To estimate bacterial C production from the Leu incorporation data would require the establishment of conversion factors specific for the studied litter colonization system. While such conversion factors are scarce for bacterial growth estimation in soil and litter (Rousk and Bååth 2011), there are many reports from aquatic ecosystems. If we would apply a widely used conversion factor from aquatic studies (3.1 ng bacterial C pmol^−1^ Leu; Cole, Findlay and Pace 1988; Rousk and Bååth 2011; Bottomley, Taylor and Myrold 2012), the 27 days of litter incubation in Experiment II resulted in bacterial C productions ranging between 4–35 μg bacterial C g^−1^ litter. Given that conversion factors were not calibrated for our experiment, these absolute values need to be interpreted with caution. However, it would appear that fungal production clearly dominated the colonization of litter in our system, resulting in several 100-fold higher fungal than bacterial production. More work would be needed to determine if this fungal dominance is common, or specific for the studied plant.

### Si release from plant litter

Our reported estimates of Si release rates from *E. arvense* (Figs 4a and d) are in range with previous estimates made from other plant litter types at similar temperature, pH and time interval (1.42–11.5 μmol Si g^−1^ day^−1^; Fraysse, Pokrovsky and Meunier 2010; Schaller and Struyf 2013). The rapid initial Si release followed by a slower long-term Si release (Fig. 4a and d) is commonly observed in batch and flow-through microcosm experiments investigating diatom (Roubeix, Becquevort and Lancelot 2008) or phytolith (Fraysse *et al.*2006a,b; Fraysse, Pokrovsky and Meunier 2010) bSiO_2_ dissolution. The two distinct stages of Si release are suggested to be contingent on the reactivity of two separate Si fractions where the faster and slower release rates are associated with small polymers and large amorphous Si aggregates, respectively (Fraysse, Pokrovsky and Meunier 2010). However, the pattern is also suggested to be intrinsic to dissolution experiments since the Si release rate would gradually slow down while approaching the equilibrium concentration as bSiO_2_ dissolution proceeds (Loucaides *et al.*^2012^).

Independent of inoculum, we observed that the N and P amendments made to microcosms (Table 1) strongly influenced total Si release (Fig. 5). One possible explanation is increasing additions of Si impurities originating from the added chemicals (KNO_3_ and K_2_HPO_4_/KH_2_PO_4_). This explanation was not supported by differences in initial Si concentrations between nutrient treatments (Table 1). Irrespective, at each C:N:P level, this would not affect the observed influence of microbes on Si release. A second possibility is increased ionic strength (mol L^−1^) of the aqueous solution with greater additions of N-, P- containing salts, which previously has been observed (Loucaides, Van Cappellen and Behrends 2008; Roubeix, Becquevort and Lancelot 2008). The marked increase in EC with higher nutrient additions (Table 2) would be consistent with this. Another possibility is that variations in pH can influence bSiO_2_ dissolution rates (Fraysse *et al.*2006a,b, 2009; Van Cappellen and Qiu 1997; Roubeix, Becquevort and Lancelot 2008). The small differences (<0.7 pH units) between live and sterile treatments are not likely to explain observed differences in Si release rates (Fraysse *et al.*2006b). We also note that both methods used to sterilize litter resulted in similar patterns of Si release from litter (Fig. 5), and the bacterial use of plant litter was identical between the two methods of sterilization (Fig. 1a).

### Effect of microbial litter decomposition on Si release

Contrary to our hypothesis (H1), we found total Si release to be reduced by ∼30%–40% in the presence of a live microbial community when compared to sterile conditions (see Fig. 5, Experiment II), and that the reduction increased with higher total microbial growth (Fig. 6c). Deviation of the ∼40% decrease at C:N:P 25:1:1 is likely explained by the attainment of chemical equilibrium for amorphous silica (1600 μM DSi at 21°C and pH 6) at sterile conditions. The association between Si release and microbial growth was further strengthened by the observation that a reduction in Si release was seen in contaminated sterile microcosms (compared to uncontaminated replicates) already at relatively low levels of microbial growth.

The reduced release of DSi under live conditions compared to sterile (Figs 5 and 6c) was unanticipated. It suggests that microorganisms either (Scenario 1) inhibit Si release from phytoliths or (Scenario 2) render released Si immobile. The latter scenario would also suggest that the actual Si release from phytoliths under live conditions could be either similar to or higher than that under sterile conditions. Elucidating the exact mechanism(s) leading to the reduction of Si released from phytoliths in the presence of a live microbial community was beyond the scope of the present study and remains unclear. However, there are several possible mechanisms that could drive these two scenarios. With regards to ‘scenario 1’, phytolith dissolution may be directly inhibited by organic metabolites produced by bacteria. This has been shown for primary silicates (Bennett and Siegel 1987; Bennett *et al.*^1988^; Ullman *et al.*^1996^; Pokrovsky *et al.*^2009^) and clay minerals (Golubev, Bauer and Pokrovsky 2006). Concerning ‘scenario 2’, released Si could be immobilized either by Si-requiring organisms (e.g. testate amoebae and diatoms) introduced to live microcosms via the added soil inoculum or by an enhanced Si precipitation in the presence of live microbes. However, Si-immobilization by these Si-acquiring organisms can be excluded in our experimental design. Filtering (1 μm) the bacterial soil inoculum in Experiment I would exclude testate amoebae (10–150 μm; Mitchell *et al.*^2000^) from the live microcosm, while growth of diatoms would be restricted due to dark incubation conditions. In addition, micrographs of plant litter collected at the experiments final day (Experiment II, data not shown) indicated no presence of testate amoebae. Another mechanism for scenario 2 could be a microbially enhanced Si-precipitation. Bioleaching of bSiO_2_ from submerged plant litter, followed by formation of Si particles upon exposure to fungi or bacteria has been observed (Bansal, Ahmad and Sastry 2006; Singh *et al.*^2008^). Organic compounds released by microbes could, theoretically, aid in the formation of organo-Si precipitates and the complexation of DSi with organic acids has been demonstrated (Marley *et al.*^1989^). Hence, clustering of organo-Si precipitates into particles >0.45 μm (see Materials and methods) would remove this Si fraction from the aqueous phase prior to DSi analysis.

### The microbial role in terrestrial Si-cycling

A microbial reduction of phytolith dissolution is contradictory to previous studies investigating the microbial influence on Si release for non-biogenic (e.g. wollastonite; Pokrovsky *et al.*^2009^) and biogenic Si material (Struyf *et al.*^2007^; Fraysse, Pokrovsky and Meunier 2010). In the studies where Si release was stimulated, this was assigned by the authors to microbial litter decomposition (Fraysse, Pokrovsky and Meunier 2010). Previous studies (Fraysse, Pokrovsky and Meunier 2010; Schaller and Struyf 2013) have shown that the release of Si and dissolved organic carbon (DOC) from submerged and decomposing plant litter is disconnected, at least during the initial stages of litter decomposition. This has been interpreted as evidence that Si release is not directly linked to decomposition of OC, which would also be consistent with the lack of a clear positive relationship between microbial activity and Si release during litter decomposition. Our results are consistent with these reports. The endpoint TOC concentrations in our microcosms corroborated the microbial growth rate and were lower with a larger reduction in Si release (Table 3). Since we did not monitor TOC changes over time, however, our resolution was limited. Also consistent with our results, higher Si release under sterile conditions has been observed previously following the addition of 5 mM NaN_3_ (microbial inhibitor) to mixed-flow reactors containing plant litter (Fraysse, Pokrovsky and Meunier 2010). The additions of NaN_3_ could have increased the ionic strength and thereby enhanced Si release rates (Loucaides, Van Cappellen and Behrends 2008). Unfortunately, no quantification of microbial activity was made in either of the studies (Struyf *et al.*^2007^; Fraysse, Pokrovsky and Meunier 2010) rendering the direct comparisons between Si release and microbial growth difficult to disentangle.

The obtained results also contrast with observations made from dissolution experiments with diatom frustules where Si release rates are enhanced by bacterial decomposition of outer organic coatings (Bidle and Azam 1999, 2001; Roubeix, Becquevort and Lancelot 2008). This suggests that the mechanism by which aquatic bacteria enhance Si release during diatom dissolution (i.e. decomposition of organic coatings) (Patrick and Holding 1985; Bidle and Azam 2001) may not be directly transferable to enhanced Si release from phytoliths during microbial decomposition of plant organic matter.

In conclusion, microorganisms had no positive influence on bSiO_2_ dissolution in our experiments, but instead resulted in a reduced release of DSi compared with sterile treatments. Assuming that our studied *E. arvense* phytoliths are representative for phytoliths in general, the microbial influence on bSiO_2_ dissolution during litter decomposition would be different from that commonly assumed. This would stand in sharp contrast to the important role of microbial decomposers in aquatic ecosystems where bacteria are shown to accelerate diatom bSiO_2_ dissolution. Since diatoms can also hold substantial reservoirs of Si in surface soils (Van Kerckvoorde, Trappeniers and Nijs 2000; Alfredsson *et al.*^2015^), the ecosystem balance of bSiO_2_ between phytoliths and diatoms need both be assessed to estimate the net contribution of microbial decomposition for the Si ‘terrestrial ecosystem filter’.
